# Mechanism and Prevention of Spiral Ganglion Neuron Degeneration in the Cochlea

**DOI:** 10.3389/fncel.2021.814891

**Published:** 2022-01-05

**Authors:** Li Zhang, Sen Chen, Yu Sun

**Affiliations:** ^1^Department of Otorhinolaryngology, Union Hospital, Tongji Medical College, Huazhong University of Science and Technology, Wuhan, China; ^2^Institute of Otorhinolaryngology, Tongji Medical College, Huazhong University of Science and Technology, Wuhan, China

**Keywords:** spiral ganglion neuron, sensorineural hearing loss, gene therapy, stem cell therapy, cochlea

## Abstract

Sensorineural hearing loss (SNHL) is one of the most prevalent sensory deficits in humans, and approximately 360 million people worldwide are affected. The current treatment option for severe to profound hearing loss is cochlear implantation (CI), but its treatment efficacy is related to the survival of spiral ganglion neurons (SGNs). SGNs are the primary sensory neurons, transmitting complex acoustic information from hair cells to second-order sensory neurons in the cochlear nucleus. In mammals, SGNs have very limited regeneration ability, and SGN loss causes irreversible hearing loss. In most cases of SNHL, SGN damage is the dominant pathogenesis, and it could be caused by noise exposure, ototoxic drugs, hereditary defects, presbycusis, etc. Tremendous efforts have been made to identify novel treatments to prevent or reverse the damage to SGNs, including gene therapy and stem cell therapy. This review summarizes the major causes and the corresponding mechanisms of SGN loss and the current protection strategies, especially gene therapy and stem cell therapy, to promote the development of new therapeutic methods.

## Introduction

Hearing loss is one of the major health problems worldwide, affecting over 5% of the population of the world or approximately 466 million people^1^. Children with hearing loss have difficulties in language and cognitive development, thus affecting their school performance, ability to integrate into mainstream job markets, and overall quality of life (Wake et al., 2004; Borton et al., 2010). Hearing loss in the elderly may increase the risk of dementia and Alzheimer’s disease (Lin et al., 2011). Based on the affected cochlear sites, hearing loss is generally categorized into conductive and sensorineural hearing loss (SNHL). SNHL is usually caused by irreversible damage to cells along the auditory pathway, including spiral ganglion neurons (SGNs). The major causes of SGN loss include harmful extrinsic (noise, ototoxic drugs, etc.) and intrinsic causes (genetic factors, aging, etc.). The mature sensorineural tissues of the cochlea in mammals, including hair cells (HCs) and SGNs, have very limited repair capacity and do not regenerate, so this damage is usually permanent.

The somata of SGNs reside in Rosenthal’s spiral canal. Each cell body of SGNs gives rise to a peripheral process that extends toward the organ of Corti and a central process that connects together to form the auditory nerve emitting into the brain, thus establishing a point-to-point communication between the cochlear HCs and the cochlear nucleus. Human SGNs are divided into two types: type I and type II afferent neurons. Ninety-five percent of the neuron population in the spiral ganglion consists of type I afferent neurons, which are myelinated and connect inner hair cells (IHC) with the cochlear nuclei of the brainstem (Eybalin, 1993). Each dendrite of type I afferent neurons innervates only one IHC, while each IHC receives contacts from 10 to 20 dendrites from type I afferent neurons (Eybalin, 1993). Type II afferent neurons account for only 5–10% of the neuron population (Spoendlin, 1972; Ruggero et al., 1982) and they are pseudounipolar and non-myelinated neurons (Berglund and Ryugo, 1986, 1991). Each type II afferent neuron innervates approximately 15 to 20 outer hair cells (OHCs), which are from the same row, while each OHC receives only one contact from one type II afferent neuron.

Until recently, the only treatments for SNHL have been hearing aids and cochlear implants, both of which are highly unnatural compared with normal sound stimulation, and they perform poorly in noisy environments. Cochlear implants are the standard therapy for severe to profound hearing loss, and their performance is variable, which is likely related to the number of residual SGNs (Seyyedi et al., 2014). No clinical therapies existed to rescue the dying SGNs or regenerate these cells once lost. Fortunately, great progress has been made in new biological therapies, such as gene therapy and stem cell therapy, providing promising perspectives for the future restoration of hearing in deaf people. This review summarizes the major causes and the related mechanisms leading to the degeneration of SGNs and discusses recent therapeutic strategies in gene therapy and stem cell therapy research to reverse SGN damage.

## Major Causes and Corresponding Mechanisms of Spiral Ganglion Neurons Loss

### Noise Exposure

Exposure to excessive levels of sound leads to a temporary threshold shift (TTS) that can fully recover to normal or a permanent threshold shift (PTS) that fails to return to pre-exposure levels. The loss of afferent ribbon synapses and the degeneration of SGNs can be triggered by noise exposure (Fernandez et al., 2015). After mild noise exposure with TTS, swelling of afferent endings and the primary degeneration of SGNs were observed in a mouse model (Puel et al., 1998; Kujawa and Liberman, 2009). During the early stage of noise exposure, the quantity and quality of the ribbon synapses significantly deceased without total recovery even after several days when the hearing was fully recovered (Shi et al., 2015). This form of damage is thought to contribute to hearing difficulties in noisy environments, tinnitus, and other auditory dysfunctions (Kujawa and Liberman, 2009). However, high-intensity exposure (>100 dB sound pressure level, SPL) or repeated overstimulation leads to PTSs (Spoendlin, 1985). After overexposure, hair cell damage can be visible within minutes, while the death of SGNs is delayed by months to years (Johnsson, 1974).

Excitotoxicity is a complex process triggered by the overactivation of glutamate receptors that results in degenerative neuronal cell death (Lai et al., 2014). Type I SGNs are activated by glutamate, and excessive release of the excitatory neurotransmitter (glutamate) from IHC could lead to the death of SGNs. Excitotoxicity is thought to play an essential role in noise-induced hearing loss. SGN afferent synapse swelling after noise exposure is likely due to glutamate toxicity (Robertson, 1983; Puel et al., 1998). Excessive glutamate release after noise overstimulation leads to the overactivation of glutamate receptors on the postsynaptic membrane of SGNs. Such overactivation leads to an influx of cations such as Na^+^ and Ca^2+^. Then, Cl^–^ and water molecules passively cross the plasma membrane, leading to edema and even death of the SGNs (Pujol and Puel, 1999; Wang et al., 2002). Administration of exogenous glutamate receptor agonists, including AMPA and kainite, to the cochlea could mimic this process (Ruel et al., 2000; Le Prell et al., 2004), while swelling of the afferent synapse could be prevented by treatment with a glutamate antagonist (Puel et al., 1998). These results suggest a contribution of excitotoxicity to SGN damage induced by noise exposure. In addition, an influx of Ca^2+^ into the afferent nerves of the cochlea leads to calcium-dependent caspase-mediated apoptosis by the intrinsic (mitochondria-mediated) pathway (Puel et al., 1998; Pujol and Puel, 1999; Ruel et al., 2007).

### Toxic Drugs

Certain therapeutic agents could cause functional impairment and cellular degeneration of SGNs. More than 130 drugs have been found to be ototoxic (Liu et al., 2011, 2012; Lanvers-Kaminsky et al., 2017). Two important classes are aminoglycoside antibiotics (Jeong et al., 2010; Wang et al., 2021) and platinum-based antineoplastic agents (Tsukasaki et al., 2000; Liu et al., 2019b,^2021^), which could cause permanent hearing loss. Cisplatin, the most commonly used platinum-based antineoplastic agent and the most ototoxic drug in frequent use in the clinic (Muggia et al., 2015), results in OHC loss in a basal to apical gradient and SGN and cell loss in the stria vascularis (Schacht et al., 2012). Degeneration of SGNs caused by toxic drugs is frequently observed secondary to hair cell loss. However, Wang et al. (2003) found that the neurotoxic effects after carboplatin treatment occurred approximately 1 day before the IHCs were injured. A unique case showed that the benefit of cochlear implantation (CI) was lost due to the use of cisplatin (Harris et al., 2011). These results indicate that SGNs are the primary injury sites after treatment with platinum-based antineoplastic agents, and they are not limited to hair cell loss. However, the mechanisms of SGN damage induced by cisplatin have not been fully explained. One of these mechanisms is thought to be mediated through ROS generation, subsequently inducing calcium influx and apoptosis (Kawai et al., 2006; Mohan et al., 2014). Cisplatin also activates apoptosis by increasing the release of cytochrome c (Garcia-Berrocal et al., 2007; Jeong et al., 2007). The expression of JNK, phospho-JNK, c-Jun, and phospho-c-Jun are also increased (Jeong et al., 2010), indicating that activation of the c-Jun N-terminal kinase signaling pathway is involved in SGN apoptosis in response to oxidative stress. Liu et al. (2019b) found that Wnt signaling activated TIGAR, protecting SGNs against cisplatin-induced damage through the suppression of oxidative stress and apoptosis. Autophagic flux was found to be activated by PRDX1 *via* the PTEN/AKT signaling pathway in SGNs after cisplatin damage, attenuating ROS accumulation to mediate protective effects (Liu et al., 2021).

### Infections

Infection with some viruses or bacteria, such as cytomegalovirus (CMV) and *Streptococcus pneumoniae*, leads to SNHL due to the degeneration of SGNs. CMV is the leading cause of congenital virus infection, and it affects around0.5–1% of all live births worldwide, with approximately 10% of infected infants developing hearing loss (Lombardi et al., 2010; Plosa et al., 2012). A histopathological study of the human temporal bone showed that the total number of SGNs was significantly reduced in ears with congenital infectious diseases compared to normal ears (Miura et al., 2002). However, the mechanisms of the pathogenesis are still unclear. Mouse models of CMV-induced profound SNHL have shown that SGNs are preferentially infected by CMV and that the number of SGNs dramatically decreases (Juanjuan et al., 2011; Schachtele et al., 2011; Bradford et al., 2015; Ikuta et al., 2015). These results indicate that a reduction in the number of SGNs may be the major cause of congenital CMV infection-induced SNHL. Increased numbers of macrophages and CD3 + mononuclear cells were detected in the SGNs of infected mice with hearing loss (Schachtele et al., 2011; Bradford et al., 2015). High levels of ROS were found to be involved in CMV-induced profound SNHL (Schachtele et al., 2011; Zhuang et al., 2018). Multiple proinflammatory molecules, including tumor necrosis factor-α, interleukin-6, CCL8, CXCL9, and CXCL10 were increased in CMV infection-induced SNHL (Teissier et al., 2011; Gabrielli et al., 2013; Melnick and Jaskoll, 2013; Bradford et al., 2015). Li et al. (2016) demonstrated that SGN apoptosis has an important relationship with SNHL induced by CMV infection. In addition to CMV, *Streptococcus pneumonia* and *Mycobacterium tuberculosis* infection-induced SNHL also led to a markedly decreased density of SGNs (Klein et al., 2003; Kuan et al., 2007; Perny et al., 2016).

### Genetic Factors

Genetic factors play a crucial role in SNHL, including congenital and later-onset hearing loss. More than 150 genes have been identified to be directly associated with SNHL. Among the genes identified are those encoding transcription factors (*POU3F4*), ion channels (*KCNQ1* and *KCNE1*), extracellular matrix components (*COCH*), cytoskeletal proteins (several unconventional myosins), and proteins of unknown function (*DFNA5*). Mutations in these genes result in either primary and/or secondary SGN damage. Primary SGN degeneration is more likely to be observed with mutations of genes that play an important role in neuronal survival and the regulation of synaptic transmission, such as *POU3F4*, *SLC17A8*, and *PJVK* (Ruel et al., 2008; Brooks et al., 2020; Cheng et al., 2020). Mutations in *POU3F4/Pou3f4*, the encoding of a transcription factor, and POU-domain protein cause deafness in humans and mice (Kandpal et al., 1996; Minowa et al., 1999). *Pou3f4*^–/–^ mice showed disrupted radial bundle fasciculation and synapse formation (Coate et al., 2012) and degeneration of SGNs (Coate et al., 2012; Brooks et al., 2020). The hair cells and supporting cells in the *Pou3f4*^–/–^ mice appeared normal, indicating that the degeneration of SGNs is primary in this mouse model. Secondary SGN degeneration often occurs due to mutations in genes affecting hair cells or supporting cells. Mutations in the *GJB2* gene, expressed in supporting cells, are the most common cause of hereditary hearing loss. Conditional Cx26-null mice exhibit secondary SGN degeneration resulting from the degeneration of hair cells and supporting cells (Wang et al., 2009; Takada et al., 2014). Degeneration of SGNs was observed in mice with mutations in other deafness genes, such as *KCNQ1* and *KCNE1* (Vetter et al., 1996; Eugene et al., 2009). In addition, hundreds of genes, including mitochondrial genes and antioxidant defense-related genes, are thought to predispose people to noise-induced, drug-induced and age-related hearing loss by aggravating SGN damage (Wang and Puel, 2018).

### Aging

Age-related hearing loss (ARHL) is the third most prevalent chronic medical condition affecting the elderly (Lethbridge-Cejku et al., 2004), and it is characterized by difficulties in speech discrimination and sound detection and localization, particularly in the presence of background noise. It is symmetric, progressive, and sensorineural, and it begins in the high-frequency region and spreads toward the low-frequency regions as age advances. SGNs are frequently lost during aging secondary to the loss of HCs (Schacht and Hawkins, 2005) as hair cells and supporting cells provide neurotrophic support, including neurotrophin-3 (NT3), brain-derived neurotrophic factor (BDNF), and glial cell line-derived neurotrophic factor (GDNF) for SGN survival (Ernfors et al., 1995; Fritzsch et al., 1997; Takeno et al., 1998). However, SGN degeneration without HC loss is common among mammals during aging. The loss of SGNs is probably independent of the age-related loss of HCs. Primary and secondary degeneration of SGNs may coincide in the same cochlea (Hequembourg and Liberman, 2001). It may be impossible to separate the primary and secondary degeneration of SGNs during the early degeneration stages of aging. Oxidative metabolism is involved in age-related SGN loss. Significant age-related loss of SGN fibers has been observed prior to HC in mice lacking copper/zinc superoxide dismutase, the first-line defense against oxidative damage caused by ROS (Keithley et al., 2005). Mitochondria play a key role in ROS generation. It has been shown that age-related loss of SGNs in mice with mitochondrial dysfunction is more severe than in control mice (Niu et al., 2007; Yamasoba et al., 2007). In addition to ROS generation, mitochondria may also promote ARHL *via* apoptosis and calcium signaling pathways.

Signaling pathways that impact the aging of the whole organism could influence age-related SGN loss, as age is the most important predictor of SGN survival. Two key molecular pathways, the insulin/insulin-like growth factor-1 (IGF-1) pathway and the lipophilic/steroid hormone pathway, are closely related to the survival of SGNs. Caloric restriction (CR) can effectively modulate the IGF-1 pathway to prevent age-related neuronal loss of the enteric nervous system (Cowen, 2002; Thrasivoulou et al., 2006). CR was found to delay auditor brainstem response (ABR) threshold shifts during aging and ameliorate SGN degeneration in mice (Park et al., 1990; Willott et al., 1995; Someya et al., 2007; Yamasoba et al., 2007). Glucocorticoids, lipophilic/steroid hormones, have been shown to have detrimental effects on neuronal function during aging (Sapolsky et al., 1986; Miller and O’Callaghan, 2005; Landfield et al., 2007). In mice lacking the β2 subunit of the nicotinic acetylcholine receptor, SGN loss was accelerated (Bao et al., 2005) and serum corticosterone (a major glucocorticoid) increased (Zoli et al., 1999) during ageing. Acceleration of age-related SGN loss was also found in mice lacking NF-κB (Lang et al., 2006) whose translocation in SGNs appears to be controlled by glucocorticoids (Tahera et al., 2006).

## Protection and Regeneration of Spiral Ganglion Neurons

Currently, there are no clinical therapies to prevent SGN degeneration or to regenerate these cells once lost. Numerous efforts have been made to explore potential therapies that could ameliorate the degeneration of SGNs. It is not surprising that agents that could interfere with the progression of SGN degeneration are promising candidates for SNHL. These pharmacological therapies include mitochondrial metabolic regulators, autophagy modulators, antioxidants or inhibitors of kinases, and apoptosis. However, there are no known drugs specifically approved by the FDA to prevent SGN degeneration or promote SGN repair. Although osmotic pumps containing neurotrophic factors (NTs) have been used to treat deaf animal models and have shown promising results, concerns about infection and the duration of efficacy restrict their widespread clinical application (Ma et al., 2019). More recent studies have focused on gene therapy and stem cell therapy, which could possibly provide long-term treatment efficacy (Liu et al., 2019a).

### Gene Therapy

Gene therapy is a method that introduces a target foreign gene or gene regulatory element into target cells to replace or fix defective genes (Mulligan, 1993). Factors including vector types, administration routes, administration time, etc., have a vital role in the treatment effect. Currently, transfer vectors include viral and non-viral vectors. The most commonly used and most promising vectors in cochlear gene therapy are adenovirus (Ad)-based and vector adeno-associated virus (AAV)-based vectors, as both have effective transduction of many cochlear cell types (Kesser and Lalwani, 2009; Ruan et al., 2010). The capacity of Ad vectors is large (26–45 kb), which greatly expands the number of target genes, while AAV vectors have limited capacity (4–5 kb). AAV vectors are not associated with any known human disease, making them unique among viral vectors. In addition to choosing a proper vector, a safe and efficient administration route is required for inner ear gene therapies. The most commonly used routes for introducing delivery vectors into the inner ear of neonatal and adult animals are through the scala media, scala tympani, and the semicircular canal (Kilpatrick et al., 2011; Gassner et al., 2012; Chien et al., 2015). Some studies also delivered viral vectors *in utero* (Bedrosian et al., 2006; Gubbels et al., 2008). Gene therapy goals for SGNs include preventing the degeneration of SGNs and promoting the regeneration of SGNs. The most studied gene therapies in animal models to protect the SGN are NTs, such as BDNF and NT3 (Wan et al., 2014; Budenz et al., 2015; Pfingst et al., 2017). Neurotrophins regulate neuronal differentiation and survival during cochlear development (Fritzsch et al., 1999; Farinas et al., 2001; Rubel and Fritzsch, 2002; Yang et al., 2011). BDNF and NT3, mainly provided by supporting cells of the organ of Corti, have important roles in the development and maintenance of SGNs (Schecterson and Bothwell, 1994; Fritzsch et al., 1999; Stankovic et al., 2004). Loss of BDNF and NT3 support leads to the gradual degeneration of SGNs (Fritzsch et al., 1999; Alam et al., 2007).

Exogenous NT (BDNF, GDNF, NT3, CNTF, and others) administration into the cochlea can prevent precipitous SGN loss (Gillespie et al., 2003) and also promote long-term survival of SGNs (Shepherd et al., 2008; Agterberg et al., 2009; Leake et al., 2011), especially when combined with electrical stimulation (Shepherd et al., 2005; Leake et al., 2013). Several studies in deaf animal models, including guinea pigs, mice (Fukui et al., 2012), rats (Wu et al., 2011) and cats, have reported improved SGN survival with virally mediated NT expression compared to controls after acoustic trauma (Table 1). Staecker et al. (1998) reported that an HSV-1 vector containing *BDNF* could almost completely rescue the damaged SGNs caused by neomycin despite the destruction of all HCs. Another study demonstrated that enhanced SGN survival was observed for up to 4 weeks in aminoglycoside/diuretic-induced deafened guinea pigs with the administration of Ad-mediated transfection of *GDNF* compared to the controls (Yagi et al., 2000). Ad-mediated gene transfer of *BDNF* and *NT3* also prevented SGN degeneration after aminoglycoside-induced deafness in guinea pigs (Wise et al., 2010). HSV1-mediated NT3 expression protected SGNs from degeneration caused by cisplatin-induced ototoxicity in aged mice (Bowers et al., 2002). In addition to SGN protection, gene therapy with NTs could also improve the survival and resprouting of the radial nerve fibers of SGNs (Shibata et al., 2010; Wise et al., 2010; Atkinson et al., 2012, 2014; Fukui et al., 2012; Chen et al., 2018).

**TABLE 1:** Studies of gene therapy for SGNs rescue in deafened animals.

Animal	Damage model	Administration route	Viral vectors	Morphological protection	References
CBA/6J mice	Neomycin	scala tympani	HSV1-*BDNF*	significantly improved SGNs survival	Staecker et al., 1998
Guinea pig	Kanamycin + ethacrynic acid	scala tympani	Ad5-*GDNF*	significantly enhanced SGNs survival	Yagi et al., 2000
Guinea pig	Kanamycin + ethacrynic acid	Scala media	AAV-*BDNF*	significantly enhanced SGNs survival	Lalwani et al., 2002
CBA/CaJ aging mice	cisplatin	scala tympani	HSV1-*NT3*	significantly improved SGNs survival	Bowers et al., 2002
Guinea pig	Kanamycin + ethacrynic acid	scala tympani	Ad-*BDNF* Ad-*CNTF*	*BDNF* alone and the combined *BDNF* and *CNTF* treatment significantly enhanced SGN survival. *CNTF* did not enhance the protective effect of *BDNF*.	Nakaizumi et al., 2004
Guinea pig	Kanamycin + ethacrynic acid	scala tympani	Ad-*BDNF*	significantly preserved SGNs in the basal turns	Rejali et al., 2007
Guinea pig	Neomycin	scala tympani	Ad-*BDNF*	higher SGNs survival and lower CI thresholds	Chikar et al., 2008
Rat	Kanamycin	scala tympani	AAV1-*GDNF*	Significantly reduced SGNs damage and improved auditory function	Liu et al., 2008
Guinea pig	Kanamycin + furosemide	scala tympani scala media	Ad5-*BDNF* Ad5-*NT3*	significant preservation of SGNs and radial nerve fiber survival	Wise et al., 2010
Rat	Noise exposure	scala tympani	Ad-*hNGF*β	Significant greater number of SGNs and smaller ABR threshold shift	Wu et al., 2011
Guinea pig	Kanamycin + furosemide	scala media	Ad5-*BDNF* or Ad5-*NT3*	Significant SGNs protection in the entire basal turn for the 1 week deaf group, in the lower basal turn for the 4 week deaf group and no protection for the 8 week deaf group	Wise et al., 2011
Mutant mice	*Pou4f3* mutant	scala media	Ad-*BDNF*	Enhanced preservation of SGNs and pronounced sprouting of nerve fiber	Fukui et al., 2012
Guinea pigs	Kanamycin + furosemide	scala media	Ad5-*BDNF* Ad5-*NT3*	Sustain protection of SGNs and directed peripheral fiber regrowth (4–11 weeks)	Atkinson et al., 2012
Guinea pigs	Kanamycin + furosemide	scala tympani	Plasmid-*BDNF*	Regeneration of SGNs neurites	Pinyon et al., 2014
Guinea pigs	Kanamycin + furosemide	scala media	Ad5-*BDNF* Ad5-*NT3*	Long term protection of SGNs (6 months)	Atkinson et al., 2014
Guinea pigs	Neomycin or Kanamycin + furosemide	scala tympani	AAV-*BDNF* AAV-*NT3*	A transient elevation in NT levels can sustain the cochlear neural substrate in the long term; BDNF was more effective than NT3 in preserving SGNs	Budenz et al., 2015
Guinea pigs	Neomycin	scala tympani	AAV2-*NT3*	Long term protection of SGNs (5–14 months)	Pfingst et al., 2017
Guinea pigs	Noise exposure	scala tympani	AAV8-*NT3*	Significant SGNs synaptic protection	Chen et al., 2018
Cat	Neomycin	scala tympani	AAV2-hBDNF AAV5-GDNF	Improved SGNs and radial nerve fiber survival	Leake et al., 2019

*SGNs, spiral ganglion neurons; HSV1, Herpes simplex virus type 1; Ad, adenovirus; AAV, adeno-associated virus; BDNF, brain-derived neurotrophic factor; GDNF, glial cell line-derived neurotrophic factor; NT3, neurotrophin-3; CNTF, ciliary neurotrophic factor.*

Noise-induced synapse loss could be prevented with inner ear gene therapy with NTs. SGN degeneration and hearing loss in rats exposed to blast waves were prevented by gene therapy with Ad-mediated human beta-nerve growth factor gene transfer (Wu et al., 2011). Synapse damage caused by noise exposure could be prevented by *Ntf3* overexpression *via* gene therapy (Wan et al., 2014). AAV-mediated *NT3* overexpression prevented and repaired noise-induced synaptopathy (Chen et al., 2018; Hashimoto et al., 2019). Pfingst et al. (2017) also demonstrated that AAV-mediated *NT3* gene therapy could prevent SGN degeneration in deafened, implanted guinea pigs.

A proper administration approach is important for SGN gene therapy. Wise et al. (2010) demonstrated that injection of vectors into the scala media resulted in more localized gene expression, greater neuron survival, and more localized fiber responses than scala tympani injection. This result indicates that vector injection into the scala media may be a better method for gene therapy of SGNs. Another important factor to consider with gene therapy is the acute treatment window. Andrew et al. showed that the efficacy of SGNs protection of viral-mediated NT expression diminished with an increasing duration of deafness, which indicates that there is a treatment window of gene therapy (Wise et al., 2011). Interestingly, Budenz et al. (2015) reported that BDNF was more effective in preventing SGN degeneration after deafness, while NT3 had a greater effect in eliciting the regrowth of radial nerve fibers. These results suggest that combining the overexpression of *BDNF* and *NT3* may have a better effect on SGN protection.

Although great progress has been made in gene therapy for protecting SGNs, there are some problems that need to be solved. NT overexpression has been reported to have detrimental effects on hearing. A recent study showed that overexpression of *Ntf3* in normal guinea pig cochleae led to disruption of synapses in the cochlea and hearing loss (Lee et al., 2016). Another study also found that overexpression of human *GDNF* in normal mice caused severe neurological symptoms and hearing loss (Akil et al., 2019). These findings indicate that extremely high levels of transgene NT expression should be avoided. Another obstacle for gene therapy application is that the gene therapy effect gradually disappears due to degeneration of the transduced cell (Atkinson et al., 2014). A long-term study on NT gene therapy showed that the efficacy of one-time injection could only last for up to 11 weeks (Atkinson et al., 2012). At 3 months after gene therapy with *BDNF* or *NT3*, peripheral auditory fibers still showed considerable regrowth in the basilar membrane area compared to the controls, although the neurotrophin levels were not significantly elevated in the cochlear fluids (Budenz et al., 2015). Finally, an important limitation blocking the transition into clinical practice is the lack of diagnostic tools to detect synaptopathy.

### Stem Cell Therapy

Cell therapy refers to the use of live cells to repair damaged cells or to replace lost cells. Stem cells and differentiated cells can be used for these purposes (Parker, 2011). Stem cells, including embryonic stem cells (ESCs), adult stem cells (ASCs), and induced pluripotent stem cells (iPSCs), are involved in the regeneration of SGNs. There are two strategies for stem cell-based therapy: to stimulate resident stem cells within the organ of Corti to differentiate into SGNs and to supply exogenous stem cells (stem cell transplantation) into the inner ear. Theoretically, the first approach is supposed to be the best strategy for repairing or replacing damaged SGNs. Unfortunately, there is an insufficient number of resident stem cells in the adult cochlea, and they are not capable of restoring hearing.

Consequently, recent studies (Table 2) have focused on the second approach (Maharajan et al., 2021). Stem cell therapy includes stem cell differentiation into target cells *in vitro* and differentiated cell transplantation into the cochlea. Gunewardene et al. (2012) proposed the stepwise differentiation of ESCs and iPSCs into otic or neuronal precursors. In our opinion, the major challenge in stem cell therapy is cell transplantation, as the environment in the cochlea is hostile to the survival of foreign stem cells. Strategies to introduce exogenous neural stem cells into the cochlea include administration *via* the perilymph and endolymph (Lang et al., 2008) into the modiolus or the cochlear nerve (Corrales et al., 2006; Ogita et al., 2009) and into the lateral wall (Zhang et al., 2013). Although transplantation into the modiolus has shown a higher cell survival rate and increased populations of exogenous cells in Rosenthal’s canal compared to transplantation into the perilymph and endolymph (Matsuoka et al., 2007; Lang et al., 2008), the transplantation process may cause hearing damage (Corrales et al., 2006; Ogita et al., 2009). The transplantation of stem cells into the sidewall of the cochlea achieved efficient results and temporary relief of auditory impairment (Zhang et al., 2013). This method may be a better choice for transplantation. Lee et al. (2017) preconditioned the scala media to reduce the potassium concentration before transplantation, thus increasing the survival of transplanted cells. However, some stem cells lose their pluripotency and differentiation ability.

**TABLE 2:** Studies of stem cell in animal models for SGN regeneration.

Animals	Damages	Type of cells	Delivery site of transplantation	Morphology change	Hearing outcome	References
Mouse	Cisplatin	mNSCs	Modiolus	Robust survival of transplant-derived cells in the modiolus of the cochlea, but the majority of grafted NSCs differentiated into glial cells	Not mentioned	Tamura et al., 2004
Guinea pig	Kanamycin and ethacrynic acid	mESCs	Modiolus	Transplanted cell was found in cochlea and project neurites toward peripheral and central nervous systems.	Significant improvement in the ABR thresholds	Okano et al., 2005
Guinea pig	Neomycin	mESCs	Scala tympani	Transplanted cells were found close both to the sensory epithelium, and the SGNs with peripheral dendritic processes projecting to the organ of Corti. Co-transplantation with mDRGs increased SGNs survival.	Not mentioned	Hu et al., 2005a
Rat	NA	mDRGs	Scala tympani	A significant difference was identified in the number of DRG neurons between the NGF and non-NGF groups. Extensive neurite projections from DRGs were found penetrating the osseous modiolus toward the spiral ganglion.	Not mentioned	Hu et al., 2005b
Guinea pig	Neomycin	mNSCs	Scala tympani	Transplanted cells expressed the neuronal marker and were found close to the sensory epithelium and adjacent to the SGNs and their peripheral processes.	Not mentioned	Hu et al., 2005c
Guinea pig	Kanamycin and frusemide	mESCs	Scala tympani	Small numbers of MESCs were detected in the scala tympani for up to 4 weeks and a proportion of these cells retained expression of neurofilament protein.	Not mentioned	Coleman et al., 2006
Gerbil	Ouabain	mESCs	cochlear nerve trunk	SGNs and neuronal processes near the sensory epithelium increased	Not mentioned	Corrales et al., 2006
Gerbil	Ouabain	BM-MSCs	Scala tympani or Modiolus	Transplanted cells were able to survive in the modiolus.	Not mentioned	Matsuoka et al., 2007
Mice and guinea pig	Noise	NSC	Scala tympani	Transplanted cells showed characteristic of both neuron tissues and the cells of the organ of Corti. SGNs increased.	Not mentioned	Parker et al., 2007
Gerbil	Ouabain	mESCs	Perilymph or endolymph	ESCs introduced into perilymph most differentiated into glia-like cells. ESCs transplanted into endolymph survived poorly.	Not mentioned	Lang et al., 2008
Guinea pig	Kanamycin and ethacrynic acid	mESCs	Scala tympani	50–75% of transplanted cells express markers of early neurons, and a majority of these cells had a glutamatergic phenotype.	Not mentioned	Reyes et al., 2008
Mouse	NA	miPSCs	Scala tympani	Neurons derived from iPS cells projected neurites toward cochlear hair cells.	Not mentioned	Nishimura et al., 2009
Rat	β-bungarotoxin	mESCs	Modiolus or internal auditory meatus	Transplanted cells were found in the scala tympani, the modiolus, the auditory never trunk. BDNF increased cell survival and neuronal differentiation.	Not mentioned	Ganat et al., 2012
Rat	Noise Neomycin	hBM-MSCs	Intravenous administration	Delivered hMSCs were largely entrapped in the lungs; Recruitment of hMSCs was limited to the spiral ganglion area	No improvement	Choi B. Y. et al., 2012
Guinea pig	Neomycin and Ouabain	hUC-MSCs	Intravenous administration	Increase in spiral ganglion and hair cells	Significant improvement in the ABR thresholds	Choi M. Y. et al., 2012
Gerbil	Ouabain	hESCs	Modiolus	Forming an ectopic spiral ganglion	improvement in the ABR thresholds	Chen et al., 2012
Rat	Ouabain	oe-NSCs	Cochlear lateral wall	NSCs migrated into RC with a high efficiency and differentiated into neurons in a degenerated SGN	Not mentioned	Zhang et al., 2013
Rat	Ouabain	mNSCs	Scala tympani	Transplanted mNSCs were more likely to differentiate into neurons in SGN-degenerated cochleae than in control cochleae	Not mentioned	He et al., 2014
Guinea pig	Neomycin	hMSCs	Scala tympani	Significant SGN increase	Not mentioned	Jang et al., 2015
Congenital deaf albino pig	Hereditary	hUC-MSCs	subarachnoid cavity	UMSC cells were detected in SGNs, basal membrane and Stria Vescularis	Detectible wave change of ABR	Ma et al., 2016
Guinea pig	Neomycin and Ouabain	hPD-MSCs	Intravenous administration	Significant SGN increase	improvement in the ABR and DOPAE thresholds	Kil et al., 2016
Rat	Noise	oe-NSCs	retroauricular approach	Oe-NSC survived and migrated around the SGNs in RC	Hearing loss was restored	Xu et al., 2016
Mouse	Neomycin	miPSCs	Scala tympani	miPSC could differentiate into hair cell-like cells and spiral ganglion-like cells	No improvement	Chen et al., 2017
Guinea pig	NA	hiPSCs	Scala tympani	The survival of transplant-derived neurons was achieved when inflammatory responses were appropriately controlled	Not mentioned	Ishikawa et al., 2017
Guinea pig	Ouabain	hESCs	Internal auditory meatus	Transplanted cells survival was poor	Partially recovered of ABR	Hackelberg et al., 2017
Mouse	NA	miPSCs	Scala tympani	Transplanted cells were observed in the cochlear perilymph, endolymph, and modiolus, and some cells expressed neural cell markers.	No improvement	Zhu et al., 2018
Guinea pig	Kanamycin and furosemide	hMSCs	Scala tympani	In deafened animals, the alginate-MSC coating of the CI significantly prevented SGN from degeneration, but the injection of alginate-MSCs only did not.	No improvement	Scheper et al., 2019a
DTR mice	DT	hESCs	Scala tympani	Transplanted hESC-derived ONP spheroids survived and neuronally differentiated into otic neuronal lineages and also extended neurites toward the bony wall of the cochlea	Not mentioned	Chang et al., 2020

*SGNs, spiral ganglion neurons; RC, Rosenthal’s canal; NA, Not Applied; mNSCs, mouse neural stem cells; mDRGs, mouse embryonic dorsal root ganglion cells; NGF, nerve growth factor; hMB-MSCs, Human mesenchymal stem cells from bone marrow; hESCs, human embryonic stem cells; rESCs, rat embryonic stem cells; mESCs, murine embryonic stem cells; hUC-MSCs, human umbilical cord mesenchymal stem cells; hPD-MSCs, human placenta mesenchymal stem cells; miPSCs, mouse induced pluripotent stem cells; oe-NSCs, olfactory epithelium neural stem cells; hPSCs, human pluripotent stem cells.*

Embryonic stem cells (ESCs) are pluripotent stem cells and have limitless potential to proliferate and differentiate. Studies have demonstrated that human ESCs (hESCs) are able to differentiate into otic neuronal progenitors (ONPs) and SGN-like cells. Newly differentiated SGN-like cells have genotypic and phenotypic SGN-specific features, and their neurites extend toward the cochlear nucleus (Matsuoka et al., 2017). Hyakumura et al. (2019) recently found that human pluripotent stem cells (hPSCs) could be derived from sensory neuronal cells, which formed synaptic connections with hair cells and cochlear nuclei in organotypic coculture. ESC-derived mouse neural progenitor cells were transplanted *via* a round window membrane into ouabain-deafened gerbils, and they successfully engrafted into the modiolus and formed ectopic ganglia with differentiated neuronal-type cells that projected to sensory cells in the organ of Corti (Corrales et al., 2006). Chen et al. (2012) demonstrated that transplantation of neural progenitors into adult ouabain-deafened animals rescued the auditory function of the deafened animals. Although hESCs have high proliferative capacity, ethical concerns, immunological rejection, difficulty in procurement, and tumorigenic potential limit their utilization.

ASCs are thought to be a promising resource for SGN regeneration from both ethical and patient compatibility perspectives. Some cell markers of stem cells in the olfactory epithelium are the same as some cells in the auditory epithelium, and they have good regenerative capacity in adults, making them a good source of SGNs (Graziadei and Graziadei, 1979; Roisen et al., 2001). Olfactory stem cells can survive and migrate to Rosenthal’s canal after transplantation (Zhang et al., 2013; Xu et al., 2016), ameliorating noise-induced hearing impairment (Xu et al., 2016). Bone marrow stromal stem cells (Naito et al., 2004) and a purified subpopulation of glial cells expressing Sox2 isolated from the auditory nerve also showed efficient cell migration and differentiation ability (Lang et al., 2015). However, ASCs showed far less differentiation ability than ESCs, which limited their application.

In recent years, much attention has been given to iPSCs with the development of reprogramming technology. iPSCs are adult differentiated cells that are genetically reprogrammed to form pluripotent stem cells. iPSCs can easily be obtained from the somatic cells of the patient. Thus, there is no concern about immunological rejection and fewer ethical problems. A research group demonstrated that hiPSC-derived neurons could form presynaptic connections with HCs in the *in vitro* coculture system (Gunewardene et al., 2016). iPSCs also have some disadvantages, such as a low proliferation rate, the tendency to differentiate into the original somatic tissue, and tumorigenicity (Nishimura et al., 2012). One main concern in regards to their tumorigenicity is the use of viral vectors during reprogramming. A recent study described a specific stepwise neural induction method for hiPSCs to eliminate undifferentiated cells from transplants, allowing the use of only terminally differentiated neurons, thus reducing the probability of tumorigenicity. First, a neural induction method was established on Matrigel-coated plates. Then, hiPSCs were differentiated into neurons on a 3D collagen matrix, and the neuron subtypes were examined. Finally, the cultured neurons were transplanted into the guinea pig cochlea (Ishikawa et al., 2017). Boddy et al. (2020) found that human-induced pluripotent cell lines are capable of differentiating into otic cell types, including hair cells and neuronal lineages, using the non-integration approach. This technology lacks genetic integration problems, making it highly attractive in the field of regenerative medicine.

## Conclusion and Future Prospects

Different approaches are being developed for the treatment of deafness. Great progress has been made in gene therapy and stem cell therapy over the last decade. There are some problems that need to be solved, including viral safety and long-term treatment effects. Ad and AAV are widely used in cochlear gene therapy, and their safety has been confirmed in animal models. Additional research should be conducted to assess the safety and efficiency of treating humans. The treatment efficacy gradually decreases over time, so a second or even regular repeated treatments may be a solution. With regard to stem cell therapy, iPSC technology is thought to be promising. As a transplant source, autologous neurons from patient-derived iPSCs are ideal for the replacement of neurons in the injured cochlea. However, some challenges need to be overcome before application to humans, such as tumorigenesis and controlled growth of transplanted cells. Future therapies to rescue auditory function must consider multiple targets.

Combining several therapeutic strategies, for instance, stem cell delivery, gene therapy and cochlear implants, may achieve better performance. The treatment effect of cochlear implants relies at least partially on the number of surviving SGNs (Yagi et al., 2000). It is not surprising that gene therapy or stem cell therapy combined with cochlear implants could enhance the performance of cochlear implants, as gene therapy and stem cell therapy could prevent SGN degeneration. Guinea pigs treated with Ad-*BDNF* had a lower CI threshold and higher survival of SGNs, indicating that the combination of Ad-*BDNF* inoculation and electrical stimulation improved the functional measures of cochlear implant performance (Chikar et al., 2008). Scheper et al. (2019a) also showed that the alginate-MSC coating of CI significantly prevented SGN degeneration. A study demonstrated that coculture with Wnt1-expressing Schwann cells enhanced the neuronal differentiation of transplanted neural stem cells (He et al., 2014). This result reminds us that cotransplantation modified cells expressing specific cytokines along with stem cells may help us overcome the barrier of a low transplant survival rate. Genetically modified hMSCs overexpressing BDNF protect neurons significantly better from degeneration than native MSCs (Scheper et al., 2019b), which indicates that genetic modification prior to stem cell transplantation may provide a better effect. Efforts should continue toward the development of gene therapy and stem cell therapy for treating deafness.

## Author Contributions

YS conceived and designed the manuscript. SC and LZ wrote the manuscript. All authors contributed to the article and approved the submitted version.

## Conflict of Interest

The authors declare that the research was conducted in the absence of any commercial or financial relationships that could be construed as a potential conflict of interest.

## Publisher’s Note

All claims expressed in this article are solely those of the authors and do not necessarily represent those of their affiliated organizations, or those of the publisher, the editors and the reviewers. Any product that may be evaluated in this article, or claim that may be made by its manufacturer, is not guaranteed or endorsed by the publisher.

## Footnotes

1
https://www.who.int/news-room/fact-sheets/detail/deafness-and-hearing-loss
